# Whole-Genome Sequence of Pseudoalteromonas sp. NC201, a Probiotic Strain for Litopenaeus stylirostris Hatcheries in New Caledonia

**DOI:** 10.1128/MRA.00477-19

**Published:** 2019-08-22

**Authors:** Louis Sorieul, Christian Rückert, Arwa Al-Dilaimi, Anika Winkler, Jörn Kalinowski, Dominique Pham, Nelly Wabete, Viviane Boulo

**Affiliations:** a Ifremer, UR Lagons, Ecosystèmes et Aquaculture Durable, Noumea, New Caledonia, France; b Laboratoire Insulaire du Vivant et de l’Environnement, Université de la Nouvelle-Calédonie, Noumea, New Caledonia, France; c Centrum for Biotechnology (CeBiTec), Bielefeld University, Bielefeld, Germany; University of Arizona

## Abstract

The marine bacterium Pseudoalteromonas sp. strain NC201 has shown probiotic potential in Litopenaeus stylirostris rearing. In this study, the complete genome of NC201 was sequenced. This genome consists of a chromosome (4.13 Mb) and a chromid (1.24 Mb). The genome contains gene clusters coding for antibacterial peptides and secondary metabolites.

## ANNOUNCEMENT

Pseudoalteromonas strain NC201 was isolated from the coastal environment of New Caledonia (1, 2). This strain showed growth inhibition against Vibrio species like the shrimp pathogens Vibrio penaeicida (3) and V. nigripulchritudo (4). NC201 displayed antibacterial activity and was tested as a probiotic for Litopenaeus stylirostris (1, 2). In order to better understand its effect on shrimp larvae, the genome of NC201 was sequenced.

A colony of strain NC201 was used to seed marine broth medium; after 16 h, 1 ml was centrifuged. DNA was extracted from the NC201 pellet and washed once with phosphate buffer, according to the conventional phenol-chloroform protocol (5), before proceeding with the sequencing. The initial sequencing of NC201 was done using an Illumina MiSeq sequencer on a TruSeq PCR-free library preparation kit as well as an 8-kbp Nextera mate pair library with a read length of 2 × 300 bp. Using Newbler v. 2.8 with standard settings, over 1 million reads (315,477,566 bp) were assembled in an initial draft sequence of 5.33 Mbp, resulting in a 59.1-fold coverage. The initial assembly consisted of 115 contigs larger than 100 bp, 65 of which were further assembled into 6 scaffolds, with an average G+C content of 43.25%. The two largest scaffolds for the two replicons that make up the genome of *Pseudoalteromonas* sp. NC201 consisted of a chromosome of 4.13 Mbp and a chromid of 1.24 Mbp. The remaining four consisted of repetitive regions like the ribosomal RNA (rrn) operons and insertion sequence (IS) elements, identified by similarity searches with BLASTN and BLASTP against the nucleotide and nonredundant databases, respectively. The initial assembly was inspected in Consed (6); repeats and single-nucleotide polymorphisms (SNPs) were resolved as far as possible based on the mate pair and paired-end data, using the “fm,” “to,” and “pr” tags attached to the read names by Newbler to assign SNPs and repeats based on reads anchored in unique contigs. This manual assembly resulted in 1 scaffold per replicon, consisting of 23 chromosome contigs and 1 chromid contig, as well as 2 small repetitive contigs.

As two of the repetitive regions were too large/complex to be bridged reliably by PCR and Sanger sequencing, a 20-kb insert library was generated according to the manufacturer’s protocol using a 2D sequencing ligation kit (Oxford Nanopore Technologies). The library was loaded as a 1/6 part of a library pool onto an R7.3 flow cell and then sequenced using a MinION Mk1 device for approximately 12 h. One- and two-dimensional base calling was performed using the MinKNOW v. 1.3.24 base caller. In total, 1,961 2D reads were obtained and were compared to the ends of contigs using BLASTN (7). Four reads were found that bridged the large repetitive regions, and they were used as guides in Consed (6) to determine the number and order of the repetitive Newbler contigs, as well as to resolve the SNPs in these elements. The final gaps, caused by either short, simple repeats or “full stops” in the Illumina data, were bridged by PCR using Phusion DNA polymerase (New England Biolabs) according to the manufacturer’s protocol with annealing temperatures between 55°C and 62°C and extension times between 15 s and 30 s. The PCR amplicons were purified using the MinElute PCR purification kit (Qiagen) according to the manufacturer’s instructions, and the DNAs were then sent for Sanger sequencing on a 3730xI DNA analyzer (Thermo) at IIT Biotech GmbH (Germany). The Sanger reads were imported into the Consed assembly project and used to close the last gaps. This approach allowed the assembly of one contig per replicon for the circular chromosome and the circular chromid. The contigs were annotated using Prokka with standard settings (8), detecting 3,441 and 1,013 coding sequences in the chromosome and chromid, respectively. The annotated sequences are available in GenBank (accession no. CP022522 through CP022523).

Analysis of the genome sequence gives us leads toward understanding the potential antibacterial activity, which may occur via short peptides synthesized as secondary metabolites (identification in eight clusters), such as bacteriocins and gramicidin/tyrocidin. The putative antibacterial activity could also be caused by synthesis of oxygen peroxide through the degradation of an amino acid by an oxidase; *lodA* and *lodB* genes were detected in the sequences, and these genes are very closely related to the amino acid oxidases found in other *Pseudoalteromonas* sp. strains that have already been identified (9, 10).

### Data availability.

The whole-genome sequence has been deposited in GenBank under accession no. CP022522 and CP022523. The SRA accession number is SRP195745.
